# Iodine and Hypothyroidism

**DOI:** 10.2174/0118715303355789250321080037

**Published:** 2025-07-04

**Authors:** Xinyi Fan, Shuang Guo, Shentao Wu, Yuan Fan, Qinghua Zhang

**Affiliations:** 1 Department of Endocrinology, First Teaching Hospital of Tianjin University of Traditional Chinese Medicine, National Clinical Research Centre for Chinese Medicine Acupuncture and Moxibustion, Tianjin, 300381, China;; 2 Yunnan University of Traditional Chinese Medicine, Kunming, Yunnan, 650500, China;; 3 Yunnan Provincial Hospital of Traditional Chinese Medicine, Yunnan, 650500, China

**Keywords:** Iodine, hypothyroidism, thyroid diseases, autoimmune hypothyroidism, goitre deficiency, mental retardation

## Abstract

Hypothyroidism is a thyroid disorder disease caused by a decrease in the synthesis or secretion of thyroid hormones and is one of the most common thyroid disorders in clinical practice. Elemental iodine is consumed daily in salt and food, such as seaweed. Iodine deficiency is the most common cause of thyroid disorders. Iodine is oxidized in the body to produce iodine-containing thyroid hormones. Iodine deficiency can lead to goitre deficiency and can also affect children and infants, leading to mental retardation and developmental delays. The causes of hypothyroidism are complex and can be divided into autoimmune hypothyroidism, post-thyroid surgery hypothyroidism, drug-induced hypothyroidism, post-^131^I therapy hypothyroidism, post-surgical pituitary or hypothalamic tumor hypothyroidism and congenital hypothyroidism. Among them, autoimmune hypothyroidism, post-thyroid surgery hypothyroidism and pharmacological hypothyroidism account for 90% of the hypothyroidism causes. Serum iodine content is very closely related to them, and in post-surgical hypothyroid diseases, iodine can also be used as a supplement to reduce the incidence of post-surgical diseases, and there exists a complementary role in the recovery of the disease. Iodine has a complementary role in the occurrence and treatment of thyroid diseases. Thus, this paper explores and summarizes the correlation between iodine and hypothyroidism around the two parts of iodine and hypothyroidism, aiming to provide a reference for daily life, clinical research and basic investigation.

## 1 INTRODUCTION

The thyroid gland is the largest endocrine organ in the body and is responsible for the formation and secretion of thyroid hormones and iodine homeostasis in the body [1]. An imbalance in the formation and secretion of thyroid hormones or iodine levels in the body results in thyroid disease. Hypothyroidism is a thyroid disorder caused by a decrease in the synthesis and secretion of thyroid hormones or a deficiency in their physiological effects. Hypothyroidism is also caused by a deficiency of transferring protein in the blood, which leads to a decrease in the body's metabolism [2]. The pathogenesis of hypothyroidism is also affected by external influences, such as aging, pregnancy, geographical area, sex, genetics, ethnicity, and history of smoking and drinking. However, the etiology is more complex [3-9]. In the United States (US), the National Health and Nutrition Examination Survey showed that the prevalence of hypothyroidism (including overt and subclinical hypothyroidism) was 4.6% [10]. The prevalence of hypothyroidism in China (including overt 1.1% and subclinical hypothyroidism 16.7%) was reported to be 17.8% [11, 12].

Iodine is an essential micronutrient that plays a vital role in metabolism. It is oxidized to produce iodine-containing thyroid hormones that control growth, metabolism, and many other bodily functions, It is essential for brain development in fetuses and newborns [13, 14]. However, iodine is a substance that the body cannot produce on its own. Thus, it must be ingested from outside sources, and both iodine overdose and iodine deficiency can cause diseases [15]. The World Health Organization suggests that an adequate iodine level for school-age children (≥ 6 years of age) and adults is a population median urinary iodine concentration of 100–199 μg/L. A population median of less than 100 μg/L indicates insufficient iodine intake. A population is considered to have severe iodine deficiency when the median urinary iodine concentration is less than 20 μg/L, moderate deficiency at 20–49 μg/L, and mild deficiency at 50–99 μg/L. A median urinary iodine concentration of < 150 μg/L in pregnant women indicates deficiency, 150–249 μg/L indicates adequate level, and ≥ 500 μg/L indicates excessive intake. A median urinary iodine concentration in lactating women and children younger than < 2 years of age of < 100 μg/L indicates deficiency, and ≥ 100 μg/L indicates adequate intake (Table **1**). Iodine is one of the most important components of thyroid hormones. Iodine deficiency can cause goiter, hypothyroidism and other diseases [16] and has a serious impact on the neurological development of pregnant women and infants [17].

In this review, we intend to summarise the literature on the association between iodine and hypothyroidism in the hope of providing references and new ideas for clinically relevant research and applications.

## 2 IODINE

Iodine is an essential trace element, also known as the intelligence element, which is vital for the development of the nervous system. Iodine is consumed through salt, drinking water [18] and food (such as kelp, fish, and other seafood rich in trace iodine [19]). In the human body, iodine is an important component for thyroid hormones [20], and in addition, studies have shown that iodine is also a strong oxidizing agent [21]. Iodine as an agent helps to improve peptic ulcers caused by *Helicobacter pylori* and some other bacterial infections by increasing the activity of immune cells [22, 23] and that it has a definite effect in fighting tumors [13, 24, 25] and enhancing innate antiviral immunity [26]. Therefore, the level of iodine intake has important effects. Too little iodine intake can lead to endemic goiter, cretinism, and hypothyroidism, while too much can lead to hyperiodized goiter, chronic lymphocytic thyroiditis, iodine hyperthyroidism and hypothyroidism [27].

### 2.1 Iodine Deficiency

Iodine deficiency is the most common cause of thyroid disorders and can result in goiter, especially affecting children and infants [28], leading to mental retardation and growth retardation [29]. Therefore, iodine is used as an herbal indicator in screening of pregnant women, as even mild iodine deficiency increases the likelihood of thyroid dysfunction [30]. Pregnant and lactating women have higher iodine requirements than other adults. The recommenced iodine intake ranges from 220 to 250 µg/day during pregnancy and from 250 to 290 µg/day during lactation [31]. Iodine deficiency in pregnant women can affect the infant's intelligence and is associated with a risk of miscarriage [32].

Currently, in our daily lives, edible salt is the main route of iodine intake in the body. In China, iodized salt is consumed to reduce the problem of iodine deficiency. Relevant studies have shown that the long-term consumption of iodized salt can effectively prevent iodine deficiency and reduce the occurrence of some thyroid dysfunction-related diseases [33]. The global implementation of salt iodization programs has reduced the incidence of iodine deficiency disorders, but 30% of the world's population remains at risk, because the agricultural soil in these geographical areas contains very small amounts of iodine [34]. Iodine metabolism in the body mostly passes through the intestinal tract to iodide, which enters the circulation and mostly accumulates in the thyroid gland or is excreted by the kidneys [35]. Excretion of iodide *via* the kidney has a serious problem in kidney dysfunction, including recurrent bacterial urinary tract infection [23, 36]. When iodine deficiency occurs, the pituitary gland increases the secretion of thyroid-stimulating hormones, which can cause thyroid dysfunction. Mild to moderate iodine deficiency causes goiter and high iodine deficiency triggers hypothyroidism [37]. Iodine deficiency is primarily associated with changes in thyroid-stimulating hormone (TSH) levels, and TSH sensitivity and age have a U-shaped correlation in individuals with elevated TSH levels [38]. Thus changes in TSH levels can be used to assess the presence of iodine deficiency and whether the disease is in remission [39].

### 2.2 Iodine Excess

High iodine intake is well tolerated by most healthy individuals [40]. However, in some individuals, excessive iodine intake may induce hyperthyroidism, hypothyroidism, goiter, and/or thyroid autoimmune disorders [41]. Thyroid dysfunction caused by excessive iodine intake is usually mild and transient, but iodine-induced hyperthyroidism can be life-threatening in some people [42]. Public health studies have shown that excess iodine can lead to lower intelligence in children [43, 44]. Excessive iodine supplementation in people in iodine-deficient areas is also one of the main causes of high urinary iodine concentrations in children [45]. This has increased the prevalence of thyroid disease in children. Thus, some parts of China have stopped supplying iodized salt to highly iodized areas [46].

Excess and deficiency of iodine affect immune system. Relevant studies have shown that excess iodine is cytotoxic, capable of damaging lymphocytes and affecting the autoimmune system [47]. This may be the main reason for the increased prevalence of autoimmune thyroiditis when iodine is consumed in excess [48]. Hyperthyroidism caused by excess iodine can affect hormone levels as well as produce an inflammatory response to other organs [49]. Excess iodine can also cause iodine toxicity, which can cause retinal damage [50], and kidney damage [51], and induce oxidative stress to destroy sperm [52].

## 3 HYPOTHYROIDISM

Hypothyroidism is a common clinical condition characterized by thyroid hormone deficiency, which may be manifested by dull expression, slow reaction, pale face, facial edema, chills, fatigue, swelling sensation of the hands and feet, weight gain, constipation and menstrual disorders in females, or there may be no obvious signs and symptoms. According to incomplete statistics, the prevalence of hypothyroidism in China is 17.8%, of which the prevalence of subclinical hypothyroidism is 16.7% and that of clinical hypothyroidism is 1.1% [12, 13]. In the United States, the National Health and Nutrition Examination Survey data showed a hypothyroidism prevalence (both 4.3% subclinical and 0.3% clinical) of 4.6% [11]. The prevalence of hypothyroidism in Colorado is 9.4%, with 9% subclinical hypothyroidism and 0.4% clinical hypothyroidism [53]. The British Thyroid Association (BTA) reported that the prevalence of hypothyroidism in the United Kingdom is approximately 3% and is 10 times more common in women than in men as they age [54]. A meta-analysis of hypothyroidism in Europe reported a prevalence of 3.05% [55]. The large differences in prevalence between countries, especially between China and the US, are due to the diagnostic criteria for TSH values in study experimental designs, with TSH levels > 4.2 mIU/L as the criterion in China, TSH levels > 4.5 mIU/L as the criterion for the National Health and Nutrition Examination Survey in the US, and TSH levels > 5.1 mIU/L as the criterion for Colorado. Differences in data between countries may also be related to the number of participants in the experiment, the age, sex, geographical area, and iodine intake of the study participants, as well as the rate of I^131^ utilized for hyperthyroidism treatment in that country or region.

**Fig. (1):**
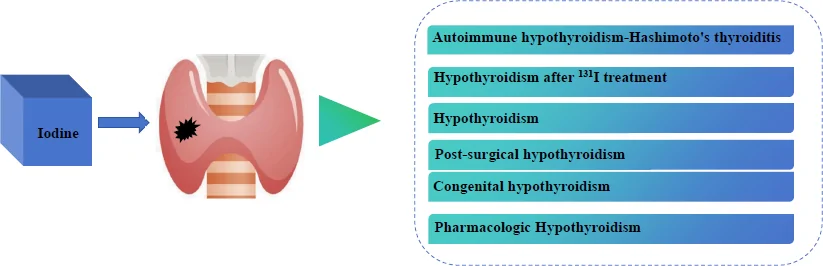
Iodine and Hypothyroidism.

The causes of hypothyroidism are complex and can be classified as autoimmune hypothyroidism, post-thyroid surgery hypothyroidism, pharmacologic hypothyroidism, post-^131^I therapy hypothyroidism, post-surgery hypothyroidism of the pituitary or hypothalamic tumors, and congenital hypothyroidism (Fig. **1**). The three major causes, thyroid surgery, hyperthyroidism due to ^131^I treatment, and autoimmunity, account for more than 90%. In patients with clinical hypothyroidism, there is a significant association between the prevalence of cancer and hypothyroidism [56-58], as well as a potential link with the development of Alzheimer's disease [59, 60].

### 3.1 Iodine and Autoimmune Hypothyroidism-Hashimoto's Thyroiditis

Hashimoto's thyroiditis (HT) is an autoimmune disease that destroys thyroid cells through cell- and antibody-mediated immune processes. It is the most common cause of hypothyroidism in developed countries [61]. Its pathogenesis is a complex process in which environmental factors, genetic predisposition, micronutrients, immune factors, cytokines, DNA, and miRNA play important roles [62]. In the treatment of HT, micronutrient supplementation and the consumption of micronutrient-rich foods can have a protective effect on the body's anti-inflammatory activity and are a major component of hormone therapy [63].

Iodine is an essential element for some important hormones in the body, such as thyroid hormone. Iodine is a substrate for thyroid hormone biosynthesis, which affects metabolism and influences the expression of genes that control various physiological functions [64]. Chronic iodine overdose induces HT [65]. Studies related to hypothyroidism in patients with HT found that when the body is immune–deficient, excess iodine leads to changes in thyroid function, increases levels of infiltrating Th17 cells in the thyroid gland and inhibits the development of T-regulatory cells [66, 67]. The data presented in a study by Liu J *et al. * suggest that excess iodine-induced reactive oxygen species (ROS) accumulation is followed by nuclear factor kappa-B (NF-κB) signaling, NLR Family Pyrin Domain Containing 3 (NLRP3) inflammatory vesicle activation, and subsequent aberrant pyroptosis in the presence of HT. Finally, interleukin (IL)-1β was released into the extracellular matrix. Decreases in ROS production, the inhibition of NF-κB signaling, or silencing of NLRP3 inflammatory vesicles inhibited NaI-induced focal death and IL-1β secretion from Thyroid follicular cells (TFCs) [68]. High iodine increases macrophage HK3 expression and promotes macrophage polarization toward M1, thereby promoting autoimmune thyroiditis [69]. Excessive iodine adsorption activates the hypoxia–inducible factor (HIF)-1α-mediated pathway, promotes the apoptosis of TFCs, and becomes an important risk factor in the development of HT [70]. In addition, it has been suggested that reduced thyroid peroxidase (TPO) mRNA may be a cause of iodine-induced hypothyroidism, which is common in patients with HT [71].

### 3.2 Iodine and Post-surgical Hypothyroidism

Post-surgical hypothyroidism includes post-thyroid surgery hypothyroidism, post-surgery hypothyroidism of the pituitary or hypothalamic tumors, and transient secondary hypothyroidism in children after cardiac surgery [72]. Among them, post-thyroid surgery is the most common type of post-surgical hypothyroidism. Studies have reported an overall incidence of post-surgical hypothyroidism in patients with normal thyroid function prior to hemithyroidectomy of 27–34% [73, 74]. Total salvage laryngectomy after radiotherapy with thyroid (hemi) resection in laryngeal cancer patients is also a high-risk factor for hypothyroidism [75]. The risk of hypothyroidism approximately doubles for every 1 IU/mL increasing the TSH levels [66]. The mechanism of postoperative hypothyroidism is not clear, but it is clear that serum TSH levels change after thyroidectomy when T4-producing tissues are significantly reduced [76]. In addition, nocturnal hypothyroidism can predict the development of central hypothyroidism after pituitary surgery [77]. Minimally invasive local nodule excision reduces the incidence of post-surgical hypothyroidism compared with thyroid lobectomy [78].

Iodine is an important element in the formation of T3 and T4 thyroid hormones. After thyroid surgery, pituitary or hypothalamic tumor surgery, the volume of T4-producing tissues and, thus, T4 levels are lowered, decreasing immune system function and facilitating recurrent infections caused by antibiotic-resistant bacteria including *Staphylococcus aureus* [79]. Iodine deficiency inhibits the Wolff-Chaikoff effect and the iodopeptide inhibition of TPO mRNA and protein synthesis, which inhibits the iodination of thyroglobulin and prevents the thyroid from synthesizing large quantities of thyroid hormone, which is not conducive to the recovery of T4 and T3 synthesis [80]. In addition, because of iodine deficiency, the patient may not be able to get rid of the inhibitory effect of high doses of iodide, thus leading to hypothyroidism.

### 3.3 Iodine and Hypothyroidism after ^131^I Treatment

^131^I therapy is an effective treatment for hyperthyroidism [81], but it can result in hypothyroidism [82]. Bhandari *et al.* [83] investigated the incidence of hypothyroidism in children with neuroblastoma treated with ^131^I-monoclonal antibody and reported that the prevalence of hypothyroidism after 2 years of treatment was 56%. Proust-Lemoine *et al.* concluded that the main pathogenesis of hypothyroidism after iodine treatment is radio-induced thyroiditis [84]. After iodine therapy, thyroid antigens are released from destroyed follicular cells and the autoimmune response is enhanced [85]. Yoshida *et al.* suggested that TSH-stimulation blocking antibody (TSBAb) activity may be the cause of hypothyroidism that occurs within a short time after ^131^I treatment [86]. Studies have shown that long-term low-dose methimazole for 60–100 months is safe and effective for treating toxic multinodular goiter, and its efficacy is not less than that of radioiodine treatment [87]. Therefore, medical practitioners can rationally choose this treatment to reduce the incidence of hypothyroidism after ^131^I treatment.

### 3.4 Iodine and Congenital Hypothyroidism

A previous study identified 42 known and novel loci affecting circulating TSH levels and 21 loci affecting circulating FT4 levels, including TPO, Forkhead box E1 (FOXE1), and Vav Guanine Nucleotide Exchange Factor 3 (VAV3) . The study suggested a genetic risk for hypothyroidism and found no evidence of gender differences in genetic variations of TSH and FT4 [88].

Persistent iodine deficiency may be a cause of hypothyroidism, which is particularly significant among pregnant women and young children of 7–9 years old [89]. Maternal thyroxine crosses the placenta before the onset of fetal thyroid function at 10–12 weeks [90]. Maternal thyroid hormones affect early brain development [91]. Low concentrations of thyroxine (T4) in pregnant women may impair fetal neurodevelopment throughout pregnancy. Iodine serves as a raw material for the synthesis of T4. A study showed that iodine treatment protects the fetal brain from the effects of iodine deficiency up to the end of the second trimester. Treatment later in pregnancy or after delivery may improve brain growth and developmental achievement slightly, but it does not improve neurologic status [92]. Therefore, in screening women for iodine micronutrient levels at the start of pregnancy is recommended, and early iodine supplementation may prevent the birth of a child with neurodevelopmental deficiencies.

### 3.5 Iodine and Pharmacologic Hypothyroidism

Because the thyroid axis readily interacts with multiple medications, the most common cause of thyroid dysfunction from drug effects is hypothyroidism [93]. The excessive use of antithyroid drugs in pregnant women is strongly associated with fetal hypothyroidism [94], inhibitors of TSH synthesis (*e.g.,* bexarotene), and the effects of hypothyroidism treatment (*e.g.,* decreases in levothyroxine absorption). The mechanism of pharmacologic hypothyroidism can be considered to be related to the inhibition of the synthesis and/or release of thyroid hormones by drugs (*e.g.* thionamides, such as iodine and iodine-containing drugs; Lithium) immune mechanisms (*e.g.* interferon-alpha (IFN-α) and other cytokines (IL-2, IL-6)), and induction of thyroiditis (increase in DIII activity), as well as tyrosine kinase inhibitors, inhibitors of TSH synthesis (*e.g.* bexarotene), and the effects of hypothyroidism treatment (*e.g.* decrease in levothyroxine absorption) [82].

Pharmaceutical doses of iodine usually result in a slight decrease in thyroid hormone levels. In patients with low thyroid reserve function, the ability to escape the Wolfe-Chaikov effect is impaired, leading to hypothyroidism [95]. The use of iodine contrast agents is also strongly associated with hypothyroidism, and studies have shown that approximately 20% of patients with no history of thyroid pathology develop thyroid dysfunction (hypothyroidism and hyperthyroidism) after receiving iodine contrast agents [96, 97]. Therefore, clinicians are advised to monitor thyroid status before and after applying that a patient is treated or examined with iodine (*e.g.,* radioactive iodine therapy (RAIT) [98]). Hoang *et al.* suggested that iodine-containing over-the-counter (OTC) medications are risky for patients susceptible to thyroid dysfunction by demonstrating that two patients with HT were treated with iodine-containing OTC medications that caused hypothyroidism, which resolved after discontinuing the medications [99]. In this regard, we advocate caution in the use of iodine-containing OTC medications in patients susceptible to thyroid dysfunction.

## CONCLUSION

Iodine is an essential trace element and one of the most important components in the synthesis of thyroid hormones. The body cannot produce iodine on its own; thus, it needs to be ingested. Hypothyroidism is a common clinical condition characterized by a lack of thyroid hormones, which may be manifested by dull expression, slow reaction, pale face, facial edema, chills, fatigue, swelling sensation of the hands and feet, weight gain, constipation, and menstrual disorders in females, *etc*.; or there may be no obvious signs and symptoms. Too much or too little iodine intake is closely related to hypothyroidism. Iodine can influence the prevalence of hypothyroidism by affecting thyroid hormone synthesis, aging, pregnancy, geographical area, sex, genetics, ethnicity, and a history of smoking and drinking. Currently, the main cause of hypothyroidism worldwide is an unequal distribution of iodine in geographical areas. Patients with hypothyroidism often need to take lifelong medications, such as levothyroxine (L-T4), which causes inconvenience and, often, psychological pressure.

Iodine is metabolized in the body mainly by conversion to iodide through the intestinal tract and then through the blood circulation, eventually accumulating in the thyroid gland or excreted from the body through the kidneys. Iodide excretion through the kidneys is affected in cases of kidney dysfunction, including recurrent bacterial urinary tract infections. Iodine is essential for the synthesis of thyroid hormones and important for neurological development, especially in fetuses and infants. The level of thyroid hormones in pregnant women affects the early brain development of the fetus. Failure to provide supplementation in cases of iodine deficiency during pregnancy may result in abnormal brain and nerve development in children. In iodine deficiency, the pituitary gland increases TSH secretion. Mild-to-moderate iodine deficiency may lead to goiter, and high iodine deficiency may lead to hypothyroidism. In cases of excess iodine, cytotoxicity occurs. Excess iodine destroys lymphocytes, affecting the immune system and increasing the incidence of immune thyroiditis.

The vast majority of hypothyroidism originates from thyroid surgery, hyperthyroidism due to treatment with ^131^I, and autoimmune disorders. In autoimmune hypothyroidism, hypothyroidism due to HT, excess iodine affects the immune system and is an important trigger for the development of autoimmune hypothyroidism. Decreases in T4-producing tissues and T4 levels after thyroid surgery and a lack of iodine will inhibit the Wolff-Chaikoff effect; thus, the iodination capacity of thyroglobulin is inhibited, which is not conducive to the synthesis of thyroid hormone and its recovery, and increases the chance of triggering hypothyroidism. In ^131^I therapy, radioactive iodine may produce hypothyroidism through radio-induced thyroiditis. Congenital hypothyroidism may be associated with insufficient iodine during pregnancy. Pharmacologic hypothyroidism is associated with pharmacologic doses of iodine or iodine contrast agents that result in slightly low thyroid hormone levels and, thus, have the potential to induce hypothyroidism.

Consequently, the level of the trace element iodine is of great significance in thyroid disease. A number of other trace elements are closely related to hypothyroidism [100]. Selenium, iron, lithium, copper and zinc are all important elements involved in the synthesis, release and metabolism of thyroid hormones [77, 101-103]. Low serum selenium levels may lead to autoimmune hypothyroidism [104]. Severe iron deficiency decreases TPO activity and interferes with thyroid hormone synthesis [105]. Lithium induces hypothyroidism by decreasing iodine uptake by the thyroid gland, leading to iodine retention [77].

In summary, it is necessary to consider the content of iodine and some trace elements in the basic research and clinical prevention and treatment of hypothyroidism. However, the pathogenesis of iodine and certain hypothyroidism is still unclear, such as hypothyroidism after thyroid surgery, and needs to be studied more deeply to provide ideas for the clinical prevention and treatment of hypothyroidism.

## Figures and Tables

**Table 1:** Relationship to iodine intake in different populations.

Indicator	Concentration cut-off values for public health significance		
Iodine Deficiency Measured by median urinary iodine concentration (ug/L)	Concentration	Iodine Intake	Iodine Status
School-age children ( ≥6 years) and adults	<20 μg/L	Insufficient	Severe deficiency
	20-49 μg/L	Insufficient	Moderate deficiency
	50-99 μg/L	Insufficient	Mild deficiency
	**100-199 μg/L**	**Adequate**	**Adequate**
	≥300 μg/L	Excessive	Risk of adverse health consequences (*e.g.* iodine- induced hyperthyroidism orautoimmune thyroid disease)
Pregnant women	<150 μg/L	Insufficient	-
	**150-249 μg/L**	**Adequate**	**Adequate iodine nutrition**
	≥500 μg/L	Excessive	-
Lactating women and children aged <2 years	<100 μg/L	Insufficient	-
	**≥100 μg/L**	**Adequate**	**Adequate iodine nutrition**

## LIST OF ABBREVIATIONS

RAIT: Radioactive Iodine Therapy
T4: Thyroxine
TPO: Thyroid Peroxidase
